# Data on people's interests related to entry into the Chinese market based on Internet activity corresponding to real-world statistical data in the period 2004–2015 in Japan

**DOI:** 10.1016/j.dib.2017.11.014

**Published:** 2017-11-04

**Authors:** Weiren Fan, Tomohisa Ueda, Yoshimasa Sagane

**Affiliations:** a Department of Business Science and Regional Development, Tokyo University of Agriculture, 196 Yasaka, Abashiri, Hokkaido 099-2493, Japan; b Department of Food and Cosmetic Science, Faculty of Bioindustry, Tokyo University of Agriculture, 196 Yasaka, Abashiri, Hokkaido 099-2493, Japan

**Keywords:** China, Japan, Google Trends, Data mining

## Abstract

This data article describes Internet activities in Japan related to entry into the Chinese market from 2004 to 2015 using data obtained from mining Google Trends. The search volumes were processed and correlated with statistical annual data on the number of Chinese companies founded by investment from Japanese companies. Relative search volumes generated by Google Trends reflect the increase and decrease in the number of Chinese companies founded by Japanese companies in the “real world.” The correlation between relative search volumes generated by Google Trends and foundation of companies in the “real world” in the study period (2004–2015) was statistically significant.

**Specifications Table**

**Table:** 

Subject area	*Management*
More specific subject area	*Business administration, International relations*
Type of data	*Graph and table*
How data was acquired	*Collected from the Google Trends web site and documentary resources*
Data format	*Raw and analyzed*
Experimental factors	*Search volume data were obtained from the Google Trends web site. Statistical data were collected from documentary resources.*
Experimental features	*Google Trends-based data and “real-world” data obtained from documentary resources were validated through correlational analyses.*
Data source location	*Japan and China*
Data accessibility	*Data are presented within this article.*

**Value of the data**

• Data based on Google Trends show good correlation with “real-world” data obtained from statistical data and can be used by scientific researchers for surveys concerning companies that enter overseas markets.
• The data can be evidence of tight correlation between “cyber-world” data obtained from web-search trends and economic behavior in the “real world,” which allows one to presume real-time behavior of the economy.
• The data can aid discussion on the relation between people's interests based on their Internet activities and the real market.

## 1 Data

**Fig. 1:**
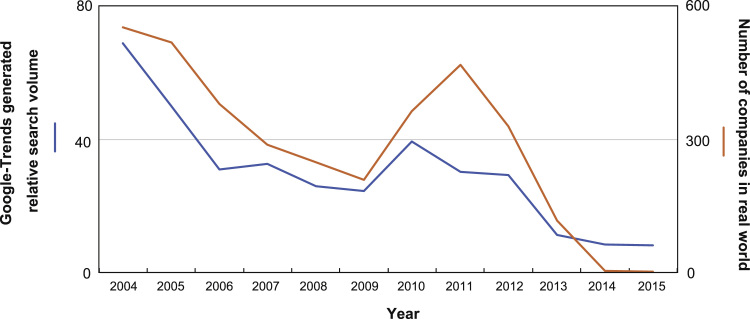
People's interest in entry into the Chinese market (Google search volume) and number of Chinese companies founded by the investment of Japanese companies during 2004–2015. For 2014–2015, the prescribed number of the companies are indicated.

**Fig. 2:**
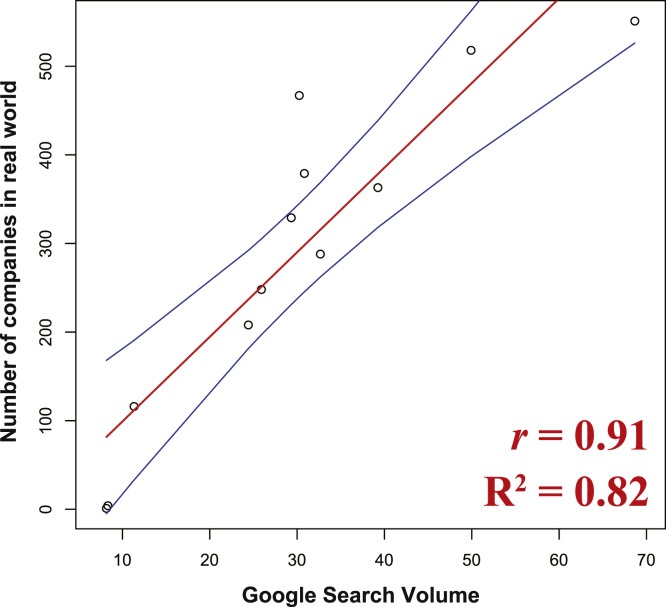
Scatter plot showing correlation between Google search volume and number of companies for each year. Linear regression (red line) is shown with 95% confidence intervals (blue lines). The coefficient of determination (*R*^2^) was 0.82. Pearson's correlation coefficient (*r*) was 0.91 with *p*-value < 0.05.

This data article provides two data. The first indicates peoples’ interests related to entry into the Chinese market based on Internet activity (“cyber world”; blue line in Fig. 1), and the second indicates the number of the Chinese companies founded by investments of Japanese companies (“real world”; orange line in Fig. 1) during 2004–2015. Scatter plots and regression statistics indicate a strong, positive linear relationship between the “cyber world” and “real world” with the coefficient of determination (*R*^2^) of 0.82, Pearson's correlation coefficient (*r*) of 0.91, and *p*-value < 0.05 (Fig. 2). Considering the cross-correlation of the data, the correlation was statistically significant only at lag 0 (Table 1).

**Table 1:** Cross-correlation analysis between the Google search volume and number of companies for each year in the period 2004–2015.

Lag	− 5	− 4	− 3	− 2	− 1	0	1	2	3	4	5
Correlation	0.27	− 0.24	− 0.55	− 0.49	0.09	0.91⁎	0.51	−0.08	− 0.43	− 0.31	− 0.07

⁎ Statistically significant with *p*-value < 0.05.

## 2 Experimental design, materials and methods

### 2.1 Design

To date, there have been four booms in Japanese companies’ investment into the Chinese market. Increase and decrease in the investment are caused by various factors, as previously discussed. Between the third (2000–2007) and fourth (2008–2013) boom periods, investments decreased significantly, probably as a reactionary fall due to monopolar concentration to the Chinese market [1]. On the other hand, even though several political conflicts between Japan and China in 2010 as well as The Great East Japan Earthquake in 2011, investments increased in 2011 [1]. Here, we compare the trends of Japanese companies’ investment into the Chinese market in the “real world” with peoples’ interests represented by their Internet activity in the “cyber world,” that is, by withdrawing online data via a search engine. Google Trends (Google Inc., Menlo Park, CA, USA; http://www.google.com/trends/), which is a freely available online tracking system of Internet hit-search volumes, was used to explore people's interests related to entry into the Chinese market. Google Trends was searched in Japan with “中国進出” (entry-to-Chinese-market) as the keyword and “Business, Industry” as the category option. “Real-world” statistical data were collected from references of the 21st Century China Research Institute [2].

### 2.2 Statistical analysis

Correlation between Google Trends-based search volumes and “real-world” statistical data about Chinese companies founded by investment from Japanese companies was assessed by the scatter plots and regression statistics using the coefficient of determination and Pearson's correlation coefficient (Fig. 2). To detect possible lag on the correlation between the “cyber-world” and “real-world” trends, cross-correlation analysis was performed. Correlations between time-lagged series (− 5 to + 5) of the “real-world” dataset and the original “cyber-world” dataset were assessed using the Pearson's correlation coefficient (Table 1). All statistical analyses were performed using the free software R (available at https://www.r-project.org) [3] and Microsoft Excel for Mac (version 15.31; Redmond, WA, USA).
